# Patients hospitalized with severe infections and hypothermia, a cohort study of mortality and prognostic factors

**DOI:** 10.1186/1757-7241-23-S1-A27

**Published:** 2015-07-16

**Authors:** Daniel P Henriksen, Christian B Laursen, Annmarie T Lassen

**Affiliations:** 1 Medical Emergency Department, OUH Odense University Hospital, Odense, Denmark; 2 Department of Respiratory Medicine, OUH Odense University Hospital, Odense, Denmark

## Background

Hypothermia is a predictor of death in patients admitted to the hospital. Although hypothermia is associated with a bad prognosis, the absolute risk in patients admitted with severe infections is unknown.

## Methods

Prospective follow-up of all patients admitted to a medical emergency department (ED) from 1 August 2009-31 August 2011. Patients were included the first time they presented with severe infection in the study period. Hypothermia was defined as a rectal temperature below 36.0°C. Patients without a measured rectal temperature at arrival were excluded. Severe infections were defined as a discharge diagnosis indicating infection as well as presence of organ failure. To assess whether hypothermia was an independent prognostic factor, we computed multivariable logistic regression analysis, adjusted for different potential confounders.

## Results

A total of 3,563 patients presenting with severe infections were included, median age 75 years (5-95% range: 33-92 years), 47.9% were males, 47.9% had Charlson Comorbidity Index>2. 147 (4.1%) presented with hypothermia. The most common site of infection among hypothermic patients with severe infection was the lower respiratory tract (83/147, 56.5%). The crude thirty-day mortality in patients with- and without hypothermia was 27.9% (95% CI: 20.8-35.9%) and 14.4% (95% CI: 13.3-15.7%) respectively. The hazard ratio for 30-day mortality adjusted for sex, age, comorbidity, and number of organ failures was 2.1 (95% CI: 1.4-3.2) compared to patients with severe infections without hypothermia.

## Conclusion

Despite only few patients admitted to a medical ED with severe infections were hypothermic at arrival, it is an important clinical finding and independent prognostic factor of short-term mortality.

